# Meiosis in crops: from genes to genomes

**DOI:** 10.1093/jxb/erab217

**Published:** 2021-08-21

**Authors:** Yazhong Wang, Willem M J van Rengs, Mohd Waznul Adly Mohd Zaidan, Charles J Underwood

**Affiliations:** 1 Department of Chromosome Biology, Max Planck Institute for Plant Breeding Research, Carl-von-Linné-Weg, Cologne, Germany; 2 University of Illinois, USA

**Keywords:** Crops, crossover, maize, meiosis, plant breeding, plant genomics, recombination, rice, tomato, wheat

## Abstract

Meiosis is a key feature of sexual reproduction. During meiosis homologous chromosomes replicate, recombine, and randomly segregate, followed by the segregation of sister chromatids to produce haploid cells. The unique genotypes of recombinant gametes are an essential substrate for the selection of superior genotypes in natural populations and in plant breeding. In this review we summarize current knowledge on meiosis in diverse monocot and dicot crop species and provide a comprehensive resource of cloned meiotic mutants in six crop species (rice, maize, wheat, barley, tomato, and *Brassica* species). Generally, the functional roles of meiotic proteins are conserved between plant species, but we highlight notable differences in mutant phenotypes. The physical lengths of plant chromosomes vary greatly; for instance, wheat chromosomes are roughly one order of magnitude longer than those of rice. We explore how chromosomal distribution for crossover recombination can vary between species. We conclude that research on meiosis in crops will continue to complement that in Arabidopsis, and alongside possible applications in plant breeding will facilitate a better understanding of how the different stages of meiosis are controlled in plant species.

## Teaser

Meiosis generates genetic diversity in natural populations and during breeding. We review meiosis research in major crop species, including an in-depth resource of cloned crop meiotic mutants and an overview of genome-wide recombination maps.

## Introduction

Meiosis is a specialized cell division that takes place during sexual reproduction and leads to the production of genetically unique haploid spores. Meiosis consists of one round of chromosome replication followed by two rounds of segregation, thereby halving the chromosome number (Mercier *et al.*, 2015; Gray and Cohen, 2016). In the first division homologous chromosomes pair, recombine, and segregate, whilst in the second division sister chromatids separate. Meiotic recombination and random segregation of homologous chromosomes combine to generate genetic variation, which is an important substrate for selection, be it natural during evolution or artificial during breeding. Notably, the first demonstration that physical recombination of chromosomes is the mechanism by which genetic linkage of traits can be broken was in maize (Creighton and McClintock, 1931), underscoring the historic role research on crops has played in the field of meiosis.

Meiosis can be broadly split into five key stages: meiotic entry, recombination initiation, chromosome synapsis, resolution of recombination intermediates, and the second meiotic division. Unlike in animals, the specification of both male and female plant germlines occurs late in development during flowering. The transition from the sporophyte phase to the gametophyte state is initiated by meiotic entry. Upon meiotic entry the replication of chromosomes and establishment of sister chromatid cohesion is followed by the association of telomeres with the nuclear envelope during the leptotene stage. Subsequently the initiation of meiotic recombination occurs via the formation of programmed meiotic DNA double strand breaks (DSBs) by a topoisomerase-like complex containing conserved SPO11 proteins. Recombination between homologous chromosomes is tightly interlinked with the lengthwise alignment of homologous chromosomes (pairing) and the formation of the synaptonemal complex (synapsis). The synaptonemal complex (SC), a protein-rich structure, connects the paired homologous chromosomes during zygotene and pachytene (the stages where recombination occurs). Meiotic DSBs are repaired by homologous recombination, which can be resolved as either a crossover (CO), which is a full reciprocal exchange, or a non-crossover (NCO), which is either a gene conversion (from the homologous chromosome) or sister chromatid-based repair. In plant species studied to date, approximately 1 in 20 meiotic DSB sites are finally repaired as a CO, suggesting selection acts to restrict CO number (Choi *et al.*, 2013; Mercier *et al.*, 2015; Sidhu *et al.*, 2015; Desjardins *et al.*, 2020). Following genetic exchange and segregation of homologous chromosomes, the second division proceeds with the release of sister chromatid cohesion, their segregation, and the emergence of haploid spores.

Meiosis is crucial for the generation of elite crop genotypes. Artificial hybridization, F_1_ hybrid propagation, and the production of recombinant F_2_ populations and/or doubled haploid populations remain key approaches in plant breeding. At the F_1_ hybrid stage, the number and position of meiotic CO events is important for the introduction of pre-breeding material into elite varieties. The application of anti-CO mutants in crop F_1_ hybrids can increase CO rates (Mieulet *et al.*, 2018); however, such mutants only increase recombination in previously recombination-competent genomic regions and this is likely insufficient to satiate the needs of crop breeders (Blary and Jenczewski, 2019). Given that recombination-suppressed regions remain suppressed in such mutants, routes to redistribute highly skewed chromosomal distributions of CO recombination remain of great interest.

In this review, we focus on monocot and dicot crop species where meiosis research is most established, and refer readers to recent reviews that have a greater focus on Arabidopsis meiosis research (Mercier *et al.*, 2015; Wang and Copenhaver, 2018). We provide a thorough resource of all previously isolated meiotic mutants of maize (*Zea mays*), rice (*Oryza sativa*), wheat (*Triticum aestivum*), barley (*Hordeum vulgare*), tomato (*Solanum lycopersicum*) and *Brassica* species (*B. rapa*, *B. oleracea*, and *B. napus*). Rice and maize are by far the most studied crop species with 64 and 22 mutants cloned, respectively (Table 1). We use this resource to highlight insights into the five keys stages of meiosis introduced above.

**Table 1.** Cloned meiotic mutants characterized in crop species

Meiotic pathway	Gene name	Rice	Maize	Tomato	Wheat	Brassica	Barley	Arabidopsis	Arabidopsis gene ID
Meiosis initiation	*AM1/SWI1/DYAD*	√	√					√	AT5G51330
	*SPL*	√						√	*AT4G27330*
	*MIL1*	√							*AT5G14070*
	*MEL1*	√							*AT2G27880*
	*MEL2*	√							No orthologue
	*AGO9/AGO104*		√					√	*AT5G21150*
	*DTM1*	√							*AT1G34640*
Sister chromatid cohesion	*SYN1/REC8/AFD1*	√	√					√	*AT5G05490*
	*SMC2*		√						*AT5G62410*
	*SMC3*		√						*AT2G27170*
	*SMC4*		√					√	*AT5G48600*
	*SCC4*		√					√	*AT5G51340*
	*SMC6*		√					√	*AT5G07660*/AT5G61460
Centromeric cohesion	*SGO1*, *SGO2*	√	√					√	*AT3G10440*/AT5G04320
DSB formation	*SPO11-1*	√	√		√			√	*AT3G13170*
	*SPO11-2*	√			√			√	*AT1G63990*
	*PRD1*	√						√	*AT4G14180*
	*PRD3/PAIR1*	√						√	*AT1G01690*
	*MTOPVIB*	√	√					√	*AT1G60460*
	P31(COMET)*/BVF1*	√						√	*AT1G03180*
	*RDR6*	√						√	*AT3G49500*
	*SDS*	√						√	*AT1G14750*
	SPO11-4*	√							No orthologue
	PHS1*		√					√	*AT1G10710*
	RR24/LEPTO1/ARR12*	√						√	*AT2G25180*
DSB processing	*MRE11*	√						√	*AT5G54260*
	*COM1*	√	√					√	*AT3G52115*
DSB repair	*RPA/RPA1a*	√						√	*AT2G06510*
	*ZYGO1*	√							*AT5G36000*/AT3G61730
	*MOF*	√							*AT1G69630*
	*ATM*	√						√	*AT3G48190*
	*RAD1*	√							*AT4G17760*
	*BRCA2*	√						√	*AT4G00020*/AT5G01630
	*RAD17*	√	√					√	*AT5G66130*
	*RAD51A*		√					√	*AT5G20850*
	*RAD51B*		√					√	*AT2G28560*
	*RAD51C*	√	√					√	*AT2G45280*
	*RAD51D*	√						√	*AT1G07745*
	*XRCC3*	√						√	*AT5G57450*
	*DMC1*	√			√		√	√	*AT3G22880*
	*FIGL1/FIGNL1*	√		√				√	*AT3G27120*
	*GEN1*	√						√	*AT1G01880*/AT3G48900
	*HOP2/AHP2*	√						√	*AT1G13330*
	*MS5*					√		√	*AT4G20900*
Synaptonemal complex	*ASY1/PAIR2*	√				√		√	*AT1G67370*
	*ASY3/DSY2/PAIR3*	√	√					√	*AT1G13330*
	*ZYP1/ZEP1*	√					√	√	*AT1G22260*/AT1G22275
	*PCH2/CRC1*	√				√		√	*AT4G24710*
Crossover formation	*MEICA1/FLIP*	√						√	*AT1G04650*
	*SUN1*	√						√	*AT5G04990*
	*SUN2*	√						√	*AT3G10730*
	*PSS1*	√						√	*AT3G63480*
	*AGG1*	√							*AT2G30480*
	*HUS1*	√							*AT1G52530*
Class I crossover	*MLH3*	√					√	√	*AT4G35520*
	*MLH1*	√						√	*AT4G09140*
	*MSH2*			√				√	*AT3G18524*
	*MSH4*	√			√	√		√	*AT4G17380*
	*MSH5*	√			√			√	*AT3G20475*
	*MSH7*				√		√	√	*AT3G24495*
	*HEI10/ZIP3*	√						√	*AT1G53490*
	*SHOC1/ZIP2*	√						√	*AT5G52290*
	*PTD1*	√						√	*AT1G12790*
	*MER3/RMC1/RCK*	√						√	*AT3G27730*
	*SPO22/ZIP4/PH1*	√			√		√	√	*AT5G48390*
	*RPA1C*	√						√	*AT5G45400*
	*RPA2C*	√						√	*AT3G02920*
	*HEIP1*	√							*AT2G30480*
	*MSH6*	√						√	*AT4G02070*
Non-crossover repair	*TOP3a*			√				√	*AT5G63920*
	*RMI1*			√				√	*AT5G63540*
	*FANCM*	√		√		√		√	*AT1G35530*
	*RECQ4*	√		√				√	*AT1G10930*/AT1G60930
Cell cycle regulator	*OSD1*	√						√	*AT3G57860*
	*RSS1*	√						√	*AT5G12360*
Chromosome segregation	*CENH3*		√					√	*AT1G01370*
	*MLKS2/SINE1*		√					√	*AT1G54385*
	*DV1/ATK1*		√					√	*AT4G21270*
	*MIS12*		√						*AT5G35520*
	*BRK1*	√							*AT2G20635*
Meiotic cytokinesis	*DCM1*	√						√	*AT1G21580*

This table consists of all the cloned meiotic mutants (and in some instances RNAi knockdown lines) as of 28 February 2021 in six crop plants (rice, maize, tomato, wheat, *Brassica*, and barley). Each cell is hyperlinked to the PubMed (https://pubmed.ncbi.nlm.nih.gov/) or Europe PMC (https://europepmc.org/) page of the original publication. Where a mutant has been characterized in Arabidopsis the original study is hyperlinked and the Arabidopsis gene is hyperlinked to TAIR (https://www.arabidopsis.org/). It should be noted this is not an exhaustive collection of all Arabidopsis meiotic mutants. Those genes marked with an asterisk have defects in DSB formation but their roles are not completely clear.

**Fig. 1.**
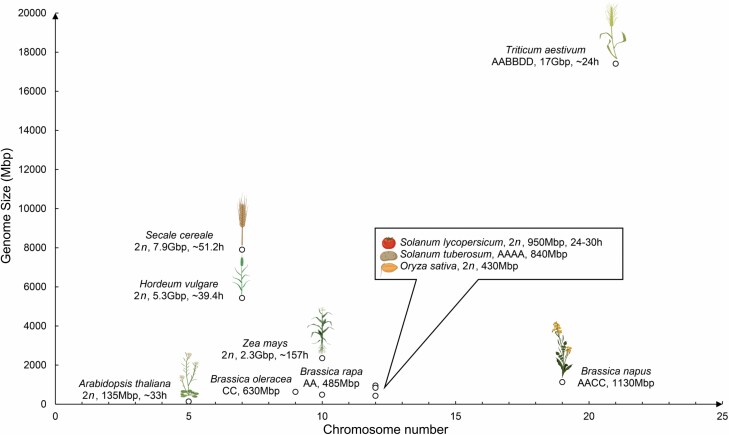
Characteristics of different plant model systems for meiosis research. The genome size, chromosome number, ploidy, and meiotic time of nine different plant species are presented. The species are Arabidopsis (*Arabidopsis thaliana*), barley (*Hordeum vulgare*), rye (*Secale cereale*), maize (*Zea mays*), tomato (*Solanum lycopersicum*), potato (*Solanum tuberosum*), rice (*Oryza sativa*), *Brassicas* (*B. oleracea*, *B. rapa*, and *B. napus*) and bread wheat (*Triticum aestivum*). Mbp, mega base pairs; Gbp, giga base pairs; h, hours. The duration of meiosis in different species is from Bennett (1977), Armstrong *et al.* (2003), Ma *et al.* (2008), and Sanchez-Moran and Armstrong (2014). Created with BioRender.com.

Crop species have highly diverse physical genome and chromosome sizes, chromosome numbers, variable ploidy, and a range of meiosis progression times (Fig. 1). In general, physical differences in chromosome length do not alter the number of meiotic COs that occur, which tends to be between one and three COs per chromosome per meiosis (Mercier *et al.*, 2015). Larger chromosomes tend to have larger regions that are suppressed for meiotic CO. We discuss how chromosomal distributions of CO are influenced by genetic and non-genetic factors, and explore genome-wide CO landscapes in those crops where such data is available. We integrate and compare the results in crops with those of Arabidopsis, which is by far the best characterized plant meiosis model. Together we make the case that pursuing meiosis research in diverse plant model systems will not only further our fundamental knowledge of meiosis, but also provide insights directly relevant for plant breeding.

## Meiotic entry: the transition to sporogenesis

In plants, the transition from the sporophyte phase to the gametophyte phase requires two distinct processes: sporogenesis and gametogenesis. Sporogenesis occurs in both male and female reproductive organs when a subset of sub-epidermal cells differentiate to become cells destined to enter meiosis—the meiocytes. Meiocytes deviate from the mitotic cell cycle and enter the meiotic cell cycle.

Genetic and physiological factors have been identified that are required for the acquisition of meiocyte cell fate in plants. The differentiation of both male and female meiocytes requires *SPOROCYTELESS* (*SPL*) in both Arabidopsis and rice (Yang *et al.*, 1999; Ren *et al.*, 2018). SPOROCYTELESS likely acts as a transcriptional repressor protein, and the mutation of *SPL* in rice leads to changes in expression of various genes involved in redox status and meiotic processes (Ren *et al.*, 2018). Notably, mutants of the gene for glutaredoxin, a redox regulator, have been identified in rice and maize that have strong defects in male sporogenesis (Hong *et al.*, 2012; Kelliher and Walbot, 2012). Related to this, increasing oxygen levels in developing maize anthers suppresses the formation of meioicytes, suggesting that a hypoxic microenvironment is important for stem cell maintenance and sporogenesis (Kelliher and Walbot, 2012).

Upon the acquisition of meiocyte cell fate, the mitotic cell cycle needs to transition to the meiotic cell cycle. This is thought to occur before the pre-meiotic S-phase when cohesion is established between the sister chromatids that are produced during DNA replication. Cohesion of chromosomes is necessary for progression through the meiotic stages, which is driven by the activity of cyclin–cyclin dependent kinase (CDK) complexes (Marston and Amon, 2004; Harashima *et al.*, 2013; Mercier *et al.*, 2015). Cohesin mediates cohesion by trapping the sister chromatids inside tripartite ring structures made up of two SMC proteins (SMC1 and SMC3) and a third α-kleisin subunit (Mercier *et al.*, 2015). Notably, sister chromatid cohesion is protected, specifically at centromeres, at the end of the first meiotic division when sister chromatids orient to a single pole. REC8, a highly conserved α-kleisin subunit, forms part of the meiotic cohesin complex, differentiating it from mitotic cohesin complexes. In Arabidopsis, maize, and rice, absence of REC8 leads to defects in sister chromatid cohesion and chromosome segregation, chromosome fragmentation at anaphase I, and sterility (Bai *et al.*, 1999; Golubovskaya *et al.*, 2006; Shao *et al.*, 2011).

A family of a meiosis-specific nuclear proteins with a conserved SMC domain play an important role in entry to a normal meiosis in maize (ZmAM1), rice (OsAM1) and Arabidopsis (AtSWI1/AtDYAD). In four strong maize *am1* alleles, including *am1-489*, meiocytes appear to enter a mitotic, rather than meiotic, division (Pawlowski *et al.*, 2009). With a weak allele, *am1-praI*, meiocytes can enter meiosis although defects are observed at the leptotene–zygotene checkpoint (Pawlowski *et al.*, 2009). The rice homologue, OsAM1, has a similar function in regulating proper chromosome structure and the leptotene–zygotene transition during early prophase I (Che *et al.*, 2011). In Arabidopsis the mutation of *AtSWI1* leads to highly reduced fertility, although viable unreduced female gametes are produced at a low frequency (Mercier *et al.*, 2003; Ravi *et al.*, 2008). Recently AtSWI1 was shown to act as an antagonist to WAPL, a factor that removes cohesin during prophase, and therefore AtSWI1 likely acts to maintain the cohesion of sister chromatids (Yang *et al.*, 2019). Overall, it appears that the role of AtSWI1 and related proteins is to maintain a suitable chromosome structure for meiotic entry and progression.

Two *meiosis arrested at leptotene* mutants (*mel1* and *mel2*) have been isolated in rice and provide important insights into the transition to meiosis and the early stages of meiotic prophase I (Nonomura *et al.*, 2007, 2011). In *mel1* mutants, germ cells are formed normally (unlike *sporocyteless* mutants) but defects are found in pre-meiotic divisions of germ cells, chromosome condensation, and meiotic progression, with arrested meiocytes having uncondensed chromosomes like those usually found at leptotene or zygotene (Nonomura *et al.*, 2007). *MEL1* encodes an ARGONAUTE protein, suggesting a link to RNAi, and is specifically expressed during sporogenesis, mediating large-scale meiotic chromosome reprogramming during the premeiosis-to-meiosis transition (Nonomura *et al.*, 2007; Liu and Nonomura, 2016). *MEL2* encodes an RNA-recognition motif (RRM) protein that appears to control the translation of meiotic RNAs, and strikingly the pre-meiotic S-phase is asynchronous in *mel2* mutants, indicating MEL2 likely functions in a separate pathway from MEL1 (Nonomura *et al.*, 2011).

Single cell sequencing has recently provided new insights into the transcriptional changes before and during meiosis in maize (Nelms and Walbot, 2019). The transition from mitosis to meiosis was not accompanied by drastic global changes in transcription, but a smooth transition in expression levels was observed. For example, two cell cycle regulator genes (the D-type cyclin genes *CycD2;1* and *CycD2;3*) that are constitutively expressed in mitotic stages are down-regulated upon the transition to meiosis (Nelms and Walbot, 2019). A more drastic change in gene expression patterns was identified during leptotene, when more than one-quarter of all transcripts changed expression level by two-fold. Maize *am1-489* and *am1-praI* mutants were also profiled and both mutants had remarkably similar transcriptional defects during meiosis, despite strong differences in chromosome behavior between the two alleles (Pawlowski *et al.*, 2009; Nelms and Walbot, 2019). In both *am1* alleles the transcriptional changes associated with the onset of meiosis were delayed, yet the meiotic transcriptional profile was eventually established, suggesting that the transcriptional landscape can be uncoupled from chromosome morphologies during the entry into meiosis.

Studies into the initiation of meiosis have been carried out in Arabidopsis, maize, and rice, yet our understanding of this process remains far from complete. Further understanding of this developmental transition could have useful applications. The artificial delay of the initiation of sporogenesis, or the transition from mitosis to meiosis, in crop species with hermaphrodite flowers (i.e. both male and female organs on the same flower) could be used in hybrid seed production. Currently, many hybrid seed production systems are based on cytoplasmic male sterility, which is genetically complex and not available in many important crops (Chen and Liu, 2014). Artificially delaying male meiosis compared with female meiosis, even by two or three days, would allow for avoidance of self-pollination and facilitate artificial hybridization (before pollen bearing anthers are produced) and hybrid seed production. In *B. napus*, different alleles of the early meiotic progression gene *MS5* represent a relevant example of the potential application of meiotic mutants in genetic male sterility breeding systems (Xin *et al.*, 2016). Overall, novel genetic, physiological, or chemical approaches that delay (male) meiosis could represent a more flexible mode of hybrid breeding.

## Recombination initiation: meiotic DNA double strand break formation and strand invasion

Meiotic recombination is initiated during the leptotene stage of meiotic prophase I by the programmed formation of meiotic DSBs, which is followed by DSB processing and repair by homologous recombination. Meiotic DSBs are produced in a huge excess compared with the final number of COs. In allotetraploid wheat, 1400 DSBs (RAD51 foci) result in 29 class I COs (HEI10 foci) across 14 homologous chromosomes; in maize, 500 DSBs (RAD51 foci) result in 16 COs (chiasma number) across 10 homologous chromosomes; and in Arabidopsis, 200 DSBs (RAD51 foci) result in 10 class I COs (MLH1 foci) across five homologous chromosomes (Choi *et al.*, 2013; Sidhu *et al.*, 2015; Desjardins *et al.*, 2020).

Meiotic recombination initiation requires key protein factors that are conserved between all eukaryotes, while co-factors to the core complex are variable between fungi, animals, and plants. In all eukaryotes programmed meiotic DSBs are generated by the type II topoisomerase-like SPO11 family of proteins. In Arabidopsis, there are three SPO11 homologues. AtSPO11-1 and AtSPO11-2 form a heterodimer and are strictly required for meiotic DSB formation, while AtSPO11-3 plays no role in meiosis but *spo11-3* mutants have somatic cell defects related to endo‐reduplication (Grelon *et al.*, 2001; Stacey *et al.*, 2006). Arabidopsis *spo11-1* mutants exhibit a classical phenotype of absence of homologous chromosome pairing and random segregation during the first meiotic division (Grelon *et al.*, 2001). MTOPVIB is a part of the catalytic core complex that can be considered as the bridge to mediate the heterodimer formation between SPO11-1 and SPO11-2, and *mtopvib* mutants do not initiate recombination (Vrielynck *et al.*, 2016). In rice, there are five SPO11 homologues. OsSPO11-1 and OsSPO11-2 are both absolutely required for fertility in rice, although *Osspo11-2* mutants still appear to form some DSBs, unlike *Osspo11-1* mutants (Fayos *et al.*, 2020). In wheat, at least one functional copy of *TaSpo11-2* is required for meiotic recombination in diploid, tetraploid, and hexaploid varieties (Benyahya *et al.*, 2020). Interestingly, different polyploid wheat varieties contain loss of function mutations in the A genome copy of *TaSpo11-2* that are absent from the diploid A-relative, suggesting the mutation occurred after polyploidization (Benyahya *et al.*, 2020). Rice *Osspo11-4* mutants do not have obvious defects during meiosis but do have a 12% reduction in fertility (by panicles), while RNAi lines of *Osspo11-4* do display meiotic defects and have a more severe reduction in fertility (An *et al.*, 2011; Fayos *et al.*, 2020). Based on the mutant analysis it appears that OsSPO11-4 does not play a major role in rice meiosis, but future analysis of high order mutants, including in *OsSPO11-3* and *OsSPO11-5*, will be instructive.

The meiotic DSB catalytic core complex also requires a number of co-factors for DSB formation to occur properly. Co-factors that play a role in DSB formation in plants include PRD1, PRD2, AtPRD3/OsPAIR1, and DFO (Mercier *et al.*, 2015). AtPRD1 acts as a key bridging factor by interacting directly with core complex members MTOPVIB, SPO11-1, SPO11-2, as well as the additional co factors DFO and PRD3 (De Muyt *et al.*, 2007; Tang *et al.*, 2017). OsPRD2 can interact with OsMTOPVIB directly and facilitates bipolar spindle construction, meiotic DSB formation, and homologous pairing (Xue *et al.*, 2019). OsPAIR1, is required for normal spindle formation and the proper establishment of homologous chromosome pairing during early meiosis (Nonomura *et al.*, 2004).

In different species, meiotic DSB formation mutants can have different phenotypes. In budding yeast, REC114 is essential for meiotic DSB formation, while the homologues in Arabidopsis and maize appear to play a role in pairing but not DSB formation (Pawlowski *et al.*, 2004; Ronceret *et al.*, 2009; Lam and Keeney, 2015). In rice the cyclin protein SOLO DANCERS (OsSDS) is essential for meiotic DSB formation, while the Arabidopsis homologue, AtSDS, is only necessary for meiotic DSB repair (De Muyt *et al.*, 2009; Wu *et al.*, 2015). In addition, two rice synaptonemal complex proteins, CENTRAL REGION COMPONENT1 (OsCRC1) and Bivalent Formation 1 (OsBVF1/OsP31^comet^), are essential for DSB formation; however, the Arabidopsis homologues AtPCH2 and AtCOMET are not required for DSB formation (Miao *et al.*, 2013; Lambing *et al.*, 2015; Ji *et al.*, 2016; Zhou *et al.*, 2017; Balboni *et al.*, 2020).

In recent years next generation sequencing has been used to map the location of meiotic DSB sites along chromosomes (Underwood and Choi, 2019). A popular approach, first developed in budding yeast, has been to exploit the covalent linkage between SPO11 and a 30–50 nt oligonucleotide that corresponds to the DSB site by immunopurifying SPO11 and sequencing the associated oligonucleotide (Pan *et al.*, 2011). This approach was successfully applied to Arabidopsis SPO11-1, where meiotic DSBs were found to be common at gene promoters, which are AT-rich and nucleosome-depleted regions (Choi *et al.*, 2018). Surprisingly, certain classes of Arabidopsis DNA transposons (*Helitron*/*Pogo*/*Tc1*/*Mariner*) are meiotic DSB hot spots while retrotransposons were depleted of meiotic DSBs (Choi *et al.*, 2018). This is consistent with SPO11-1 acting as an ‘opportunistic’ enzyme in open chromatin. In contrast to meiotic DSB maps in Arabidopsis, mice, and yeast, maize RAD51 ChIP-seq revealed that meiotic DSBs can form in all chromosomal regions including retrotransposons (He *et al.*, 2017; Underwood and Choi, 2019). In mice it has recently been shown by ChIP-seq that DMC1 binds to regions very close to the DSB site while RAD51 binds to adjacent regions, and as such this may have implications for the interpretation of the maize RAD51 ChIP-seq data (He *et al.*, 2017; Hinch *et al.*, 2020). In the future, genome-wide analyses on meiotic recombination initiation, ideally by orthogonal approaches (e.g. SPO11-oligo sequencing, DMC1 ChIP-seq, RAD51 ChIP-seq), in a wider array of plant species will provide insights into the roles of meiotic recombination in plant genome evolution. Related to this, it will be interesting to explore whether DSB interference, a phenomenon described in budding yeast that reduces the possibility of two DSB events occurring close to one another, is conserved in plant species (Garcia *et al.*, 2015).

Following meiotic DSB formation, DSBs are processed and then repaired by homologous recombination. DSBs are first processed by the MRN complex (MRE11, RAD50, and NBS1) and COM1 to release SPO11 proteins, facilitating exonuclease end resection to produce the 3′ ssDNA overhangs (Ji *et al.*, 2013; Luo *et al.*, 2014; Mercier *et al.*, 2015; Wang and Copenhaver, 2018; Wang *et al.*, 2018). The highly conserved BRCA2 can recruit DMC1 and RAD51 (two RecA-related recombinases) onto the ssDNA to facilitate homologous recombination (Wang and Copenhaver, 2018). DMC1 is specifically expressed in meiotic cells and is absolutely required for meiotic CO (Wang and Copenhaver, 2018). Many DSB repair mutants lead to meiotic chromosome fragmentation but Arabidopsis and rice plants lacking DMC1 have univalent meiotic chromosomes (Pradillo *et al.*, 2012; Mercier *et al.*, 2015; Wang *et al.*, 2016; Wang and Copenhaver, 2018). DMC1 is essential for synapsis in both Arabidopsis and rice, but unlike Arabidopsis *dmc1* mutants, homologous chromosome pairing can occur in rice plants lacking DMC1 (Wang *et al.*, 2016). Additionally, in hexaploid wheat (*Triticum aestivum*) it appears that a specific *DMC1* gene located on the D genome is required for the stabilization of chromosome pairing at low and high temperatures (Draeger *et al.*, 2020).

Directing meiotic DSBs to genomic locations where they do not usually occur could be a useful technique in plant breeding. The presence of a meiotic DSB at a given genomic location is a prerequisite for recombination but does not ensure a recombinant outcome, as meiotic DSBs can equally be repaired by sister chromatid-based repair. Directing meiotic DSB formation will likely require the recruitment of SPO11-1, SPO11-2, and MTOPVIB, and potentially other factors, to specific genomic locations. Meiotic DSBs are of a very specific molecular nature: a covalent linkage between the catalytic tyrosine residue of SPO11 proteins and the 5′ terminus of the broken DNA strand leaves a free 3′ hydroxyl group (Pan *et al.*, 2011). Therefore, SPO11-induced breaks generate a unique molecular signature, which appears distinct to endonuclease enzymes like Cas9, and this likely facilitates further processing by the MRN complex and COM1, as outlined above. Fusion proteins between dCAS9 (for targeting) and MTOPVIB have been recently tested in Arabidopsis, and although they do not lead to targeted meiotic DSBs, they can complement *mtopvib* mutants (Yelina *et al.*, 2021, Preprint). In the future, alterations of such an approach, or simultaneous modulation of meiotic DSB interference, may allow for the direct targeting of meiotic DSB events in plant hybrids to expedite introgression breeding.

## Chromosome synapsis: the formation of the synaptonemal complex

The formation of the synaptonemal complex (SC) occurs in tandem with meiotic DSB formation and strand invasion. The SC is a proteinaceous connection between the homologous chromosomes and is made up of three main elements: axial/lateral elements (AE/LE), transverse filaments, and the central element (Mercier *et al.*, 2015; Wang and Copenhaver, 2018). The two axes act as a scaffold that is zipped together by the transverse filaments and plays an important role in the maturation of meiotic recombination intermediates to facilitate CO events. During recombination initiation, the meiotic chromatin is arranged in loops that are anchored at the underlying axes. Meiotic DSBs are thought to occur on the loops, and a subset of DSBs are then recruited to the axis for recombination with homologous chromosomes. This special relationship between DNA loop and chromosome axis is called the ‘tethered loop–axis’ model and is based on findings in yeast and mice (Kleckner, 2006). However, the key proteins that mediate interactions between chromatin loops and AEs remain to be identified in plants. In plants, synapsis and SC formation requires meiotic DSB formation in rice, maize, and Arabidopsis. SC formation is defective in rice *sds* and *mre11* mutants, which have respective defects in DSB formation and processing (Ji *et al.*, 2013; Wu *et al.*, 2015). Likewise rice and maize mutants in the *RAD51C* gene, which is required for strand invasion, do not form a SC (Kou *et al.*, 2012; Jing *et al.*, 2019). The reliance of SC formation on DSBs in crops is found to be in line with reports in Arabidopsis (Mercier *et al.*, 2015; Wang and Copenhaver, 2018).

Axial element proteins mark the two homologous chromosomes during the first stages of prophase I. Before synapsis, the chromatin is diffuse, and sister chromatid cohesion is maintained by cohesion complexes that include REC8, which is itself an axis marker. After synapsis of homologous chromosomes, axial elements are described as lateral elements. ASY1, a HORMA domain protein, was initially described in Arabidopsis and *B. oleracea* as an AE (Armstrong *et al.*, 2002). During early prophase I, ASY1 marks the diffuse chromatin, and later, from leptotene to pachytene, it exclusively marks the axes of synapsed chromosomes, where it gradually disappears (Armstrong *et al.*, 2002). Mutation of *PAIR2* in rice, the *AtASY1* orthologue, leads to 24 completely unpaired univalents at pachytene and diakinesis, suggesting a key role in homologous chromosome pairing and synapsis (Nonomura *et al.* 2004). Likewise, wheat RNAi lines that knockdown *TaASY1* expression have reduced synapsis during prophase I (Boden *et al.*, 2009). In rye, ASY1 is exclusively located at AE/LEs in both A and B chromosomes where, unlike orthologous proteins in Arabidopsis and barley, it remains at the SC until its disintegration (Hesse *et al.*, 2019). In autotetraploid potato, ASY1 staining has shown patches of higher and lower intensity compared with diploid potato indicating a difficulty in axis maintenance during prophase I, and also demonstrated possible switching of synapsis partners (Choudhary *et al.*, 2020). Rice *pair3* mutants revealed a new AE/LE factor that was later shown to be conserved in Arabidopsis, where the orthologue, AtASY3, interacts directly with ASY1 (Yuan *et al.*, 2009; Ferdous *et al.*, 2012). PAIR3, a coiled-coil domain protein, is associated with both unsynapsed AEs and synapsed LEs, and is required for pairing, recombination, and SC assembly (Wang *et al.*, 2011*a*).

The transverse filament plays the role of bridging the homologous axes of the SC, and the central element is positioned along the center of the SC. In rice, the ZEP1 protein has been identified as a central element protein (Wang *et al.*, 2010). The *ZEP1* gene was identified using a reverse genetics approach, based on the earlier study of *ZYP1* genes in Arabidopsis, where two recently duplicated genes have made double mutant analysis difficult (Higgins *et al.*, 2005). Interestingly, in the rice *zep1* mutant, SC assembly does not occur, yet the chromosomes can align along their entire lengths and an increased number of COs are observed despite reduced seed set (Wang *et al.*, 2010). Increased CO formation has also recently been reported in Arabidopsis *zyp1* null mutants (Capilla-Pérez *et al.*, 2021; France *et al.*, 2021). A second transverse filament protein identified in rice is CENTRAL REGION COMPONENT1 (CRC1) (Miao *et al.*, 2013). In *zep1* mutants CRC1 is not located on chromosomes, while in *crc1* mutants ZEP1 is not found on meiotic chromosomes (Miao *et al.*, 2013). In contrast to rice *zep1* mutants, *crc1* mutants are sterile (Miao *et al.*, 2013). Notably the meiotic roles of CRC1 and the Arabidopsis homologue PCH2 appear to be quite different. In rice CRC1 is required for DSB formation, loading of PAIR2/ASY1, and SC formation, while in Arabidopsis PCH2 is not required for normal axis formation, although defects in ZYP1 polymerization are observed (Miao *et al.*, 2013; Lambing *et al.*, 2015). In *B. rapa*, PCH2 is required for normal ASY1 loading and axis remodeling (Cuacos *et al.*, 2021). *Braissica rapa pch2* mutants (10 chiasmata per meiocyte) have significantly reduced COs compared with wild-type controls (17 chiasmata per meiocyte) (Cuacos *et al.*, 2021). Cuacos *et al.* (2021) also demonstrated an important role for PCH2 in pericentromeric CO formation in both Arabidopsis and *B. rapa*. In future, analysis of *zep1/zyp1* and *crc1/pch2* mutants in a wider number of plant species, as well as the cloning of classical synapsis mutants in maize and tomato mutants (Havekes *et al.*, 1994; Pawlowski *et al.*, 2003), will likely provide further answers on the role of the SC in meiosis.

The pairing and synapsis of homologous chromosomes is a unique feature of meiosis. In the plant species studied to date, meiotic DSBs play an important role in SC formation. Yet, in budding yeast and *Drosophila*, the SC can be formed in a DSB-independent manner (Bhuiyan and Schmekel, 2004; Joyce and McKim, 2009; Tanneti *et al.*, 2011). It remains to be seen whether DSB-independent SC formation can occur naturally in plants or even if this could be achieved in an engineered fashion. For instance, the pairing of homologous chromosomes in the absence of CO formation could be used to generate non-recombinant reverse breeding populations as it could facilitate balanced segregation of homologous chromosomes that occurs rarely in non-recombinant mutants like *spo11-1* (Grelon *et al.*, 2001; Wijnker *et al.*, 2012). On the other hand, forcing the synapsis of, and CO between, homoeologous chromosomes from divergent species could be useful for introducing wild germplasm in crop breeding programs.

## Resolution of recombination intermediates: to crossover or not to crossover?

Meiotic DSBs are repaired by homologous recombination with the homologous chromosome or the sister chromatid. Recombination intermediates formed between homologous chromosomes can be resolved, and genetically detected, either as CO or non-CO (also known as gene conversion) recombinant products. In Arabidopsis, maize, and wheat, only 5% of repair events are through meiotic CO (Choi *et al.*, 2013; He *et al.*, 2017; Desjardins *et al.*, 2020). At least one meiotic CO, the so called ‘obligate’ CO, is required per homologous chromosome pair per meiosis to ensure that correct chromosome segregation occurs in meiosis I (Martini *et al.*, 2006). Meiotic COs generate a new combination of alleles, which increases genetic diversity in gametes (Youds and Boulton, 2011).

There are two types of meiotic CO pathways—the class I and class II CO pathways. In Arabidopsis and rice, the majority of CO events occur via the class I pathway, which accounts for 80–90% of total CO events (Higgins *et al.*, 2004; Mercier *et al.*, 2015). Class I COs depend on the activity of ZMM proteins (named after the budding yeast proteins Zip1-4, Mer3, Msh4, and Msh5), which are proposed to protect joint molecule recombination intermediates. For instance, purified human MSH4/5 heterodimers, otherwise known as the MutSγ complex, bind and stabilize double Holliday junctions (Lynn *et al.*, 2007). Class I COs are subject to CO interference, which means that CO events are spread apart more than would occur at random, yet the mechanism of CO interference is still not fully understood (Lynn *et al.*, 2007).

Many class I CO mutants have been studied in Arabidopsis and various crops (Higgins *et al.*, 2004; Mercier *et al.*, 2015). Rice *mer3* mutants are completely sterile with an obvious reduction in bivalent formation (12 bivalents in wild-type, 5 bivalents in *mer3*) and chiasmata frequency (20.8 in wild-type, 5.8 in *mer3*) (Wang *et al.*, 2009). Rice *msh5* mutants are similarly sterile but exhibit fewer chiasmata (2.10) per meiocyte than *mer3* mutants (Luo *et al.*, 2013). Fewer chiasmata per meiocyte were also reported in rice *msh4* (1.71) and *msh4/msh5* (1.76) double mutants compared with other ZMM mutants, suggesting the MutSγ complex may play an earlier role to stabilize progenitor Holliday junctions in rice (Zhang *et al.*, 2014). In allotetraploid *B. napus*, the reduction of functional copies of *MSH4* genes prevents non-homologous COs suggesting that stabilization of allopolyploid meiosis can be facilitated by the loss of functional copies of MutSγ genes (Gonzalo *et al.*, 2019). In allotetraploid wheat (*Triticum turgidum*), the MutSy complex maintains ~85% of all COs and is required for the obligate chiasma. Intriguingly, much like in *B. napus*, in allopolyploid wheat the loss of function of MutSy genes (*MSH5B* in allotetraploid wheat; *MSH5B* and *MSH4D* in allohexaploid wheat) has been described, suggesting a possible adaptive role in the control of CO (Desjardins *et al.*, 2020).

MLH1 and HEI10 are two important proteins for the formation of class I COs and mark chiasmata on late pachytene chromosomes. MutL-homologue (MLH) proteins play a crucial role in the DNA mismatch repair (MMR) pathway, and in eukaryotes the MLH1–MLH3 complex has been co-opted for the resolution of double Holliday junctions as meiotic COs. In tomato, MLH1 foci mark 70% of late recombination nodules and their distribution suggests they mark strongly interfering COs (Lhuissier *et al.*, 2007). Spontaneous exonic deletion in the barley *MLH3* gene led to a 50% reduction in chiasmata, consistent with the 39% reduction in chiasmata in the Arabidopsis *mlh3* mutant (Colas *et al.*, 2016). Barley *mlh3* mutants are also defective in synapsis, which was not previously observed in the Arabidopsis *mlh3* mutant (Colas *et al.*, 2016). In rice, *Osmlh3* (14.9 chiasmata per cell) and *Osmlh1* (15.2 chiasmata per cell) mutants have relatively modest reductions in chiasmata frequency (19.5 chiasmata in wild-type) (Mao *et al.*, 2021). This reduction is far less than for rice ZMM mutants (Zhang *et al.*, 2014), suggesting other proteins may be able to act as class I CO resolvases in rice (Mao *et al.*, 2021). Unlike MLH1 foci, which first appear at late pachytene stage to mark class I CO foci, HEI10 is first found on meiotic chromosomes at synapsed regions before later specifically co-localizing with MLH1 on class I CO foci. Rice *hei10* mutants form 6.5 chiasmata per cell, and those chiasmata appear to lack CO interference, while early recombination events and SC formation is normal (Wang *et al.*, 2012). In rice the HE10 Interaction Protein 1 (HEIP1) was found via a yeast two-hybrid screen for HEI10 interaction partners. *heip1* mutants are sterile and HEIP1 interacts directly with ZIP4 and MSH5, and is proposed as a novel ZMM protein (Li *et al.*, 2018).

The class II COs account for around 10–20% of total COs and do not exhibit interference (Berchowitz *et al.*, 2007). Class II COs do not rely on the MutSy complex but require the action of MUS81, which has been implicated in the resolution of class II COs in Arabidopsis, but mutants have yet to be studied in any crop species (de los Santos *et al.*, 2003; Berchowitz *et al.*, 2007; Olivier *et al.*, 2016). In tetraploid wheat, an antibody raised against TaMUS81 demonstrated that four MUS81 foci were formed per cell in *mutSγ* loss of function mutants, consistent with about 12% of CO coming from class II (Desjardins *et al.*, 2020).

There is emerging evidence that meiotic CO genes can influence pairing and meiotic CO between homoeologous chromosomes in polyploid plant species. Mutants of the wheat ZMM gene *TaZIP4-B2*, located within the *Pairing homoeologous 1 (Ph1)* locus, led to increased homoeologous CO in crosses with related wild species, suggesting ZIP4 gene dosage may influence the choice between homologous and homoeologous CO (Rey *et al.*, 2017). Meanwhile, the wheat DNA mismatch repair protein MSH7-3D was identified as a key inhibitor of homoeologous recombination and its loss is likely responsible for the classic *Pairing homoeologous 2* (*Ph2*) mutant phenotype (Serra *et al.*, 2021). In allopolyploid *B. napus*, the *Pairing Regulator in B. napus* (*PrBn*) locus on chromosome 9 (C genome) was identified as a controlling factor of homoeologous chromosome pairing in haploid *B. napus* (Jenczewski *et al.*, 2003; Liu *et al.*, 2006). Remarkably, using a different population and approach, Higgins *et al.* (2021) identified a QTL in a syntenic region of chromosome 9 (A genome) that controls homeologous recombination in *B. napus* allotetraploids. The identification of the causal gene at this locus will illuminate the molecular mechanisms of the stabilization of allopolyploid meiosis in *B. napus*.

The majority of meiotic DSBs are not repaired by CO repair pathways but by non-CO repair (Gray and Cohen, 2016; Xue *et al.*, 2018). In Arabidopsis, the protein products of *FANCM* (Crismani *et al.*, 2012), *RECQ4* (Schröpfer *et al.*, 2013; Séguéla-Arnaud *et al.*, 2015), and *TOP3α* (Séguéla-Arnaud *et al.*, 2015) have all been implicated in distinct non-CO repair pathways. Mutants in the aforementioned genes were retrieved from ZMM mutant suppressor screens, and their mutation led to an increase in CO via the class II pathway, resulting in increased fertility. Simultaneous mutation of multiple anti-CO pathways (e.g. *fancm recq4* double mutants and *figl1 recq4* double mutants) can further increase CO rate in inbred and hybrid Arabidopsis compared with the single pathway mutants (Fernandes *et al.*, 2018). Mutants in anti-CO genes have been reported in some crop species. Cultivated tomato *recq4* mutants are fertile and have a 2.7-fold increase in CO recombination (Mieulet *et al.*, 2018). Similarly, in *recq4* mutant interspecific tomato hybrids between *S. lycopersicum* and *S. pimpinellifolium*, CO increases were observed by ring bivalents and SNP marker genotyping of F_2_ progeny at respective rates of 1.54- and 1.8-fold (de Maagd *et al.*, 2020). Further to this, rice and pea (*Pisum sativum*) mutants for *FANCM* and *RECQ4* genes have increased CO rates (Mieulet *et al.*, 2018). *Brassica rapa* and *B. napus fancm* mutants also have increased CO rates at 3.0- and 1.3-fold, respectively (Blary *et al.*, 2018). Mutants in *FIGL1*, which caused a 1.8-fold increase in meiotic CO rate in Arabidopsis hybrids, are sterile in rice, pea, and tomato, demonstrating the potential for altered fertility of anti-CO mutants in different plant species (Girard *et al.*, 2015; Mieulet *et al.*, 2018).

## Genomic analysis of crossover distribution

In plants, as in other eukaryotes, meiotic COs are not uniformly distributed in the genome. CO hot spots recombine at a rate higher than the genome average, while cold spots do not recombine (Mézard *et al.*, 2015; Mercier *et al.*, 2015; Choi and Henderson, 2015). COs mostly occur within gene-rich areas, close to gene promoter and terminator regions, where AT-rich DNA motifs are enriched and nucleosome occupancy is low (Wijnker *et al.*, 2013; Choi *et al.*, 2013; Marand *et al.*, 2017, 2019; Kianian *et al.*, 2018; Rommel Fuentes *et al.*, 2020). Recombination cold spots are observed in heterochromatin-rich centromeres and telomeres in most plant species (Si *et al.*, 2015; Marand *et al.*, 2017; Kianian *et al.*, 2018; Gardiner *et al.*, 2019; Rowan *et al.*, 2019; Rommel Fuentes *et al.*, 2020).

**Fig. 2.**
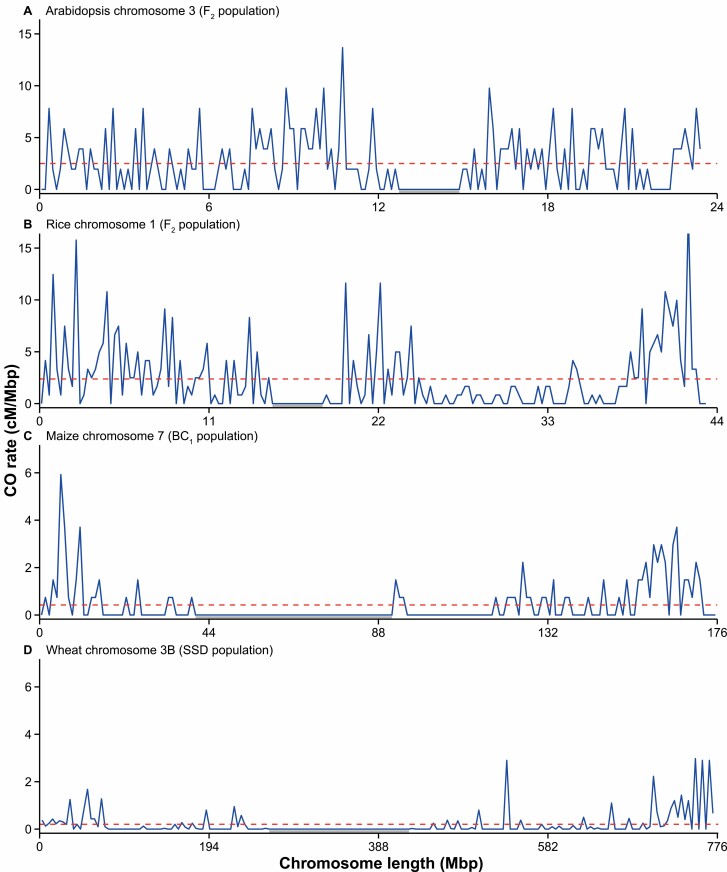
Chromosomal crossover distributions in Arabidopsis, rice, maize, and wheat. (A) cM/Mbp on Arabidopsis chromosome 3. *Arabidopsis thaliana* Col-0 *×* L*er* F_2_ population with 192 individuals from published sequencing data (Choi *et al.*, 2016) followed by calling of CO locations using TIGER (Rowan *et al.*, 2015), 0.1333 Mbp sliding window. (B) cM/Mbp on rice chromosome 1. *Oryza sativa* ssp. *japonica* cv. Nipponbare *× O. longistaminata* F_2_ population with 241 individuals selected from published genotyping data (Furuta *et al.*, 2017), 0.25 Mbp sliding window. (C) cM/Mbp on maize chromosome 7. *Zea mays* ssp*. mays* cv. B73 × (B73 × Mo17) male BC_1_ population with 135 individuals from published sequencing data (Kianian *et al.*, 2018), 1 Mbp sliding window. (D) cM/Mbp on wheat chromosome 3B. SSD population derived from a *Triticum aestivum* L. Chinese Spring × Renan cross with 305 individuals from published genotyping data (Choulet *et al.*, 2014), 4 Mbp sliding window. The grey shading beneath each chromosome represent the centromeres, which we genetically defined as the contiguous windows lacking COs that flank published centromere co-ordinates from each species (Cheng *et al.*, 2002; Wolfgruber *et al.*, 2009; Choulet *et al.*, 2014; Underwood *et al.*, 2018). The red dashed lines represent the mean cM/Mbp value for each chromosome. Plots were produced using ggplot2 within R version 4.0.3.

We selected four meiosis model species (Arabidopsis, rice, maize, and wheat) with varying genome sizes (Fig. 1) and compared CO landscapes of representative chromosomes (Fig. 2) (Choulet *et al.*, 2014; Choi *et al.*, 2016; Furuta *et al.*, 2017; Kianian *et al.*, 2018). Representative chromosomes were chosen from Arabidopsis, rice, and maize based on the physical size and the distribution of CO events on the chromosomes fairly reflecting all chromosomes within that species (Si *et al.*, 2015; Kianian *et al.*, 2018; Underwood *et al.*, 2018). Wheat chromosome 3B was included due to the public availability of CO data (Choulet *et al.*, 2014). In order to illustrate the distribution of CO events, we plotted CO rate (in cM/Mbp) per physical position on the chromosome and normalized for physical chromosome length leading to a visual chromosomal landscape of meiotic COs.

Arabidopsis subtelomeres and pericentromeric regions are areas with higher recombination than the genome average (Underwood *et al.*, 2018; Rowan *et al.*, 2019). On Arabidopsis chromosome 3, the CO rate is relatively evenly distributed along the chromosome arms (Fig. 2A). The centromeric region (13.5–14 Mbp) completely lacks COs, while the highest cM/Mbp values are found adjacent to the centromere (at ~11 Mbp) (Fig. 2A). In Arabidopsis, a positive correlation between meiotic DSB levels and meiotic CO levels is found at the broad scale (Choi *et al.*, 2018). COs do not form in inverted and translocated regions, while mutants that lose non-CG DNA methylation have increased COs in centromeric regions (Zapata *et al.*, 2016; Underwood *et al.*, 2018).

The rice genome is relatively compact compared with other monocot crop species, and this results in a CO distribution that is more similar to Arabidopsis than to maize and wheat (Figs 1, 2B) (Si *et al.*, 2015). On rice chromosome 1 (Fig. 2B), the highest cM/Mbp values are found in the distal regions (0–3 and 40–43 Mbp) and adjacent to the centromere (~20–25 Mbp) (Fig. 2B). Like in Arabidopsis, historical recombination events in rice are associated with gene promoters and terminators (Choi *et al.*, 2013; Marand *et al.*, 2019). More than 80% of the historical recombination events occurred in 5.3% and 4.2% of the whole genome sequence in *indica* and *japonica* rice subspecies, respectively, demonstrating the existence of CO hot spots (Marand *et al.*, 2019). However, relatively few hot spots are conserved between the two subspecies despite sharing a common ancestor 400 000 years ago. In both subspecies, specific DNA transposon classes (*PIF*, *Harbinger*, *Stowaway*) and also simple sequence repeats were over-represented within CO hot spots, while retrotransposon classes are depleted (Marand *et al.*, 2019).

In contrast to Arabidopsis and rice, plant species with higher repeat contents tend to have large interstitial and centromeric regions that are devoid of CO, and the majority of CO events occur in distal euchromatic regions (Choulet *et al.*, 2014; Demirci *et al.*, 2017; Marand *et al.*, 2017; Kianian *et al.*, 2018; Dreissig *et al.*, 2019; Rommel Fuentes *et al.*, 2020). A clearly polarized chromosomal distribution of CO is observed for maize (Fig. 2C) and wheat (Fig. 2D). In both species, cM/Mbp values are elevated in the distal subtelomeric regions, compared with much lower values in the interstitial and centromeric regions. On maize chromosome 7, COs mainly occur in the subtelomeric euchromatin (Fig. 2C), correlating to higher gene densities (Schnable *et al.*, 2009; Kianian *et al.*, 2018). In maize, COs are restricted to less than 10% of the physical genomic length, and therefore vast interstitial and centromeric regions are devoid of CO in all maize chromosomes (Fig. 2C) (Kianian *et al.*, 2018; Luo *et al.*, 2019). Mirroring Arabidopsis and rice, maize COs are generally excluded from retrotransposons, despite reported meiotic DSBs in these elements (He *et al.*, 2017; Kianian *et al.*, 2018). Gene conversion events have been observed in maize centromeres, where it is predicted to be widespread and may contribute to centromere evolution (Shi *et al.*, 2010). On wheat chromosome 3B, the CO rate is variable within the interstitial regions, showing locally elevated rates, although the average CO rate in these regions is substantially lower compared with the subtelomeric regions (Fig. 2D). In wheat, two simple DNA motifs (A-stretch and CCG) and two DNA transposon-related motifs (from *TIR-Mariner* and *CACTA* elements) correlate with CO rate at the whole genome level (Darrier *et al.*, 2017). Similar to maize and wheat, polarized CO landscapes are observed in other crop species, such as tomato (Demirci *et al.*, 2017; Rommel Fuentes *et al.*, 2020), potato (Marand *et al.*, 2017), and barley (Higgins *et al.*, 2012; Dreissig *et al.*, 2019)

Multiple factors have been demonstrated to affect the distribution of CO events, and these can be simply separated into genetic and non-genetic factors. Genetic polymorphism between homologous chromosomes is largely seen as inhibitory to CO, since sequence divergence inhibits homologous recombination, as exemplified by introducing polymorphism at yeast CO hot spots (Borts and Haber, 1987). Classical examples include large genetic inversions that prevent pairing, synapsis, and ultimately CO (Anderson *et al.*, 2010; Zapata *et al.*, 2016). Other structural variants (including translocations, insertions, and deletions) are also likely inhibitory to CO formation, while copy number variants and transpositions could lead to non-allelic homologous recombination (Zapata *et al.*, 2016; Underwood and Choi, 2019). A relevant example is the comparison of the CO landscapes of melon and cucumber, two *Cucumis* species that diverged 10 million years ago. In cucumber, meiotic CO frequency is relatively constant along the chromosomes, whereas melon, which experienced an expansion of LTR class I TEs after divergence, has a distalized CO landscape where the expanded pericentromeric regions are suppressed for meiotic CO (Morata *et al.*, 2018). Similar differences in CO positioning have been found between onion (distalized CO positioning) and welsh onion (more proximal CO positioning), although the basis of this is not understood (Albini and Jones, 1987). In general, large CO cold spots are observed in species with larger chromosome sizes (Fig. 2), and consistent with this, centromere size has a linear relationship with genome size (Zhang and Dawe, 2012).

The relationship between genetic polymorphism and meiotic CO appears to be more complex than increasing genetic polymorphism leading to increased meiotic CO suppression. For instance, in Arabidopsis intermediate SNP densities associate positively with CO rate before a threshold is reached and a negative association is found, suggesting a non-linear relationship (Blackwell *et al.*, 2020). Genetic factors can also control CO distribution in *trans*, for instance in *Brassica* where the A genome CO rate is redistributed to pericentromeric regions in AAC triploids compared with AA controls (Pelé *et al.*, 2017). This phenomenon is most apparent when certain A and C genomes are combined suggesting a potential QTL could underlie this redistribution effect.

Alongside genetic control of CO distribution, DNA methylation, histone modification, nucleosome occupancy and DNA accessibility, DNA replication timing, dosage of meiotic proteins, sex and 3D chromosomal confirmation have all been implicated in the control of CO distribution (Giraut *et al.*, 2011; Higgins *et al.*, 2012; Choi *et al.*, 2013, 2018; Ziolkowski *et al.*, 2017; Underwood *et al.*, 2018; Lambing *et al.*, 2020; Golicz *et al.*, 2020). The interactions of genetic and non-genetic control of CO distribution in crop species will likely be further unraveled in the coming years.

## Maintenance of sister chromatid cohesion and the second meiotic division: the emergence of haploid gametes

After COs have been resolved, homologous chromosomes segregate to opposite poles during anaphase I. In meiosis I cohesion is maintained at centromeres due to maintenance of cohesin complexes, and this facilitates the two sister chromatids of one homologue orienting towards the same pole. During anaphase II, the kinetochores of sister chromatids are oriented towards opposite poles and the previously protected centromeric cohesin is released. Release of the centromeric cohesion allows for proper segregation of sister chromatids and eventually the formation of haploid gametes at the end of meiosis II (Mercier *et al.*, 2015).

In yeast, metazoa, and plants, shugoshin, literally ‘guardian spirit’ in Japanese, is required for the protection of centromeric cohesion complexes from cleavage by separase (Watanabe, 2005). The maize shugoshin mutant *Zmsgo1* precociously separates sister chromatids’ centromeres during telophase I, leading to infidelity of chromosome segregation during meiosis II and complete sterility (Hamant *et al.*, 2005). ZmSGO1 centromeric localization is dependent on REC8, whereas OsSGO1 localization is independent of REC8 but does require OsAM1 (Hamant *et al.*, 2005; Wang *et al.*, 2011*b*). In rice, Os*sgo1* mutants also fail to maintain cohesion at centromeres during meiosis I, and the centromeric localization of OsSGO1 depends on the main spindle checkpoint kinase, Bub1-related kinase 1 (BRK1) (Wang *et al.*, 2011*b*, 2013).

Like mitosis, progression through the meiotic stages is driven by the activity of cyclins–CDKs, which phosphorylate universal cell cycle proteins and meiosis specific factors (Marston and Amon, 2004; Harashima *et al.*, 2013; Mercier *et al.*, 2015). CDKA1 has peak activities at both metaphase I and metaphase II, suggesting it is required for progression through these important meiotic stages (Dissmeyer *et al.*, 2007; Bulankova *et al.*, 2010). However, continuation to anaphase and exit from the division phase requires a decrease in CDK activity, which is achieved by the anaphase-promoting complex/cyclosome (APC/C) targeting cyclins for degradation (Marston and Amon, 2004; Pesin and Orr-Weaver, 2008; Harashima *et al.*, 2013; Mercier *et al.*, 2015). Thus, a fine-tuned cyclin–CDK activity is required for the transition from meiosis I to meiosis II. However, molecular players involved in this regulation, which mostly modify the cyclin–CDK–APC/C module, appear to be poorly conserved across eukaryotes (Mercier *et al.*, 2015).

Arabidopsis mutants of genes that play a role in the cyclin–CDK–APC/C module have been shown to skip the second meiotic division and give rise to unreduced gametes (Mercier *et al.*, 2015). *OMISSION OF SECOND DIVISION1* (*OSD1*), encoding an APC/C inhibitor, is required for meiosis II entry in both Arabidopsis and rice (d’Erfurth *et al.*, 2009; Cromer *et al.*, 2012; Mieulet *et al.*, 2016). Both Arabidopsis and rice *osd1* mutants undergo only the first meiotic division leading to the production of unreduced male (100% penetrance in Arabidopsis and rice) and female (85% penetrance in Arabidopsis and 91% penetrance in rice) gametes (d’Erfurth *et al.*, 2009; Mieulet *et al.*, 2016). TARDY ASYNCHRONOUS MEIOSIS (TAM) is a type A cyclin (CYCA1;2), essential for the entry to meiosis II in Arabidopsis (d’Erfurth *et al.*, 2010). Arabidopsis *tam* null mutants undergo the first meiotic division leading to the production of unreduced gametes in both male (∼90% penetrance) and female (∼30% penetrance) lineages (Bulankova *et al.*, 2010; d’Erfurth *et al.*, 2010). Specific mutants in Arabidopsis *THREE DIVISION MUTANT1* (*TDM1*), which is close to a conserved CDK phosphorylation site, terminate at the end of meiosis I and produce diploid gametes, whereas *tdm1* loss of function mutants fail to exit meiosis and enter a third aberrant division (Cifuentes *et al.*, 2016).

Hybrid vigor contributes to the high yield of commercial seeds and could be fixed through seeds by synthetic apomixis (Birchler *et al.*, 2006; Khanday *et al.*, 2019). The combination of mutants that skip the second meiotic division with those that do not initiate meiotic recombination (e.g. *spo11-1*) or establish sister chromatid cohesion (e.g. *rec8*) leads to the ‘mitosis instead of meiosis’ (*MiMe*) genotype (d’Erfurth *et al.*, 2009; Mieulet *et al.*, 2016). *MiMe* was first demonstrated in Arabidopsis where self-fertile *spo11 rec8 osd1* triple mutants produce non-recombined, unreduced eggs that can be fertilized by non-recombined, unreduced sperm, to generate fully hybrid tetraploid offspring (d’Erfurth *et al.*, 2009). *MiMe* can be an important element in the realization of synthetic apomixis in crop species (d’Erfurth *et al.*, 2009). Mirroring the work in Arabidopsis, rice *osd1 pair1 rec8* triple mutants produce non-recombined diploid male and female gametes and give rise to tetraploid offspring (Mieulet *et al.*, 2016). Notably the *osd1 pair1 rec8* triple mutant shows varied fertility (from full fertility to highly reduced fertility) in different rice genetic backgrounds, suggesting possible modification by genetic or environmental factors (Mieulet *et al.*, 2016; Wang *et al.*, 2019). The combination of *MiMe* with the engineering of embryo development in the absence of a paternal genetic contribution, the second element of synthetic apomixis, has been recently reported in rice via parthenogenesis (egg cell specific expression of *OsBBM1*) and haploid induction (mutation of *OsMTL*) (Khanday *et al.*, 2019; Wang *et al.*, 2019). Apart from *osd1* mutants in rice, mutants that modify the meiotic cell cycle are largely underexplored outside of Arabidopsis, and therefore reverse genetic approaches will likely help resolve the function of known genes in various crop species. Forward genetic screens for genes involved in unreduced gamete formation in crops, alongside the isolation of known genetic factors in *Brassica* and potato (Jongedijk *et al.*, 1991; Mason *et al.*, 2011), may identify novel meiotic (cell cycle) regulators.

## Conclusion

The core function of meiosis is to recombine chromosomes and reduce the chromosome number by half, and it is therefore essential to the process of sexual reproduction because it derives haploid cells from diploid cells (Mercier *et al.*, 2015). Meiotic recombination and reduction division are key aspects of vertical models of genetic transfer as well as the evolution of sex (Bell, 1993). Unsurprisingly, the core protein factors involved in meiotic DSB formation, synaptonemal complex formation, and meiotic CO are known to be conserved between fungal, animal, and plants species. Despite the conservation of the meiotic process through eukaryotic life, there are notable variations, including the evolution of the meiotic DSB hot spot specifying factor PRDM9 in vertebrates, and the complete loss of class I CO pathways in fission yeast cells (Mercier *et al.*, 2015; Grey *et al.*, 2018). Modifications of the meiotic process also occur in plant species. For instance, natural alleles of the Arabidopsis *HEI10* gene control meiotic CO rate *in trans* (Ziolkowski *et al.*, 2017) while in wheat a novel *RECQ7* gene was shown to be important for meiotic gene conversion despite its having been lost in many plant species including Arabidopsis (Gardiner *et al.*, 2019). Starkly, Arabidopsis *rmi1* mutants cannot progress in meiosis due to chromosome fragmentation and formation of chromatin bridges (Chelysheva *et al.*, 2008; Hartung *et al.*, 2008), while tomato *rmi1* mutants have no meiotic defects (Whitbread *et al.*, 2021). For most plant biologists, Arabidopsis will always be the ground truth, or gold standard, yet embracing a wide panel of model species is clearly valuable because each species has followed a distinct evolutionary trajectory, leading to unique epistatic contexts.

In the coming years various topics and approaches are likely to play a role in the better understanding of plant meiosis. The interaction between meiosis and the environment appears to be an emerging topic, especially in a time of global climatic variation. Plants are sessile organisms that cannot determine their environment, and meiosis is an important, and potentially vulnerable, aspect of plant reproduction. Already in Arabidopsis and barley, temperature has been demonstrated to alter meiotic CO rates (Higgins *et al.*, 2012; Phillips *et al.*, 2015; Lloyd *et al.*, 2018). Diploid gamete formation has also been demonstrated to be partially controlled by temperature (de Storme *et al.*, 2012). The interaction of meiotic processes with temperature and other abiotic and biotic stresses will be important to establish whether meiotic CO recombination can be adaptive under stress conditions. Emerging technologies including live cell imaging of meiosis, long read sequencing, and single cell genomics will improve the resolution by which meiosis can be observed visually and genetically (Prusicki *et al.*, 2019; Sun *et al.*, 2019).

Meiosis research in crop species is intrinsically linked with crop improvement and breeding, as meiotic recombination is a key substrate of breeding. The application of approaches that can increase CO rate and change CO distribution can be useful in crop breeding for increasing genetic gain per breeding cycle. The approaches discovered so far have been tested in a limited number of plant species and genetic contexts. Future research will no doubt unravel whether anti-CO and epigenetic mutants, which respectively modify the absolute number and distribution of COs, can have similar or different effects in genetic backgrounds with varying levels of genetic polymorphism. This will help identify if there are particular levels, or types, of genetic polymorphism where modulation of CO level and/or position can lead to negative effects including chromosomal rearrangements or reduced fertility. On the other hand, harnessing the *MiMe* system and the application of synthetic apomixis in more crop species could represent a route to fixing hybrid vigor in commercial seed production.

Above and beyond the useful applications of meiosis in plant breeding, using crops as model systems has become much more accessible to academic researchers due to the developments in genome editing and genomics. Using crop species strengthens our understanding of meiosis and should not be obligatorily categorized as applied research. Indeed, we expect that rapid cycling crop varieties that are amenable to generate mutants by CRISPR/Cas9 may be useful not only for reverse genetic studies but as a means for generating mutants for suppressor and/or enhancer screens. As outlined, we propose that meiosis research in Arabidopsis and other plant model systems will continue to complement one another and lead to the discovery of novel genes, processes, and mechanisms that are involved in meiosis.
